# 1139. Immunogenicity and Safety Study of a Quadrivalent Meningococcal Conjugate Vaccine (MenACYW-TT) Compared to a Meningococcal Reference Vaccine (MCV4-TT) in Healthy Adolescents

**DOI:** 10.1093/ofid/ofad500.980

**Published:** 2023-11-27

**Authors:** Javier Díez-Domingo, Róbert Simkó, Giancarlo Icardi, Chan Poh Chong, Céline Zocchetti, Olga Syrkina, Siham Bchir, Isabelle Bertrand-Gerentes

**Affiliations:** FISABIO, Valencia, Comunidad Valenciana, Spain; Futurenest Clin. Res. Ltd, Miskolc, Borsod-Abauj-Zemplen, Hungary; University of Genoa, San Martino Policlinico Hospital, Genoa, Liguria, Italy; KTP-National University Children's Medical Institute, National University Hospital, Singapore, Not Applicable, Singapore; Sanofi, Lyon, Auvergne, France; Sanofi, Lyon, Auvergne, France; Sanofi, Lyon, Auvergne, France; Sanofi, Lyon, Auvergne, France

## Abstract

**Background:**

MenACYW-TT (MenQuadfi^®^) is a quadrivalent meningococcal conjugate vaccine licensed for use in individuals ≥ 12 months age in the EU and certain other countries and ≥ 2 years of age in the US. Safety and immunogenicity of a single dose of MenACYW-TT were evaluated in 10-17 years old healthy adolescents vs a quadrivalent (MCV4-TT [Nimenrix^®^]) meningococcal vaccine licensed ex-US. Co-administration of MenACYW-TT with 9vHPV and Tdap-IPV vaccines was also studied.

**Methods:**

In this modified double-blind, Phase-3 study (NCT04490018) conducted in Hungary, Italy, Spain, and Singapore, meningococcal vaccine-naïve adolescents or MenC-primed (when < 2 years of age) were randomized to receive either MenACYW-TT or MCV4-TT and co-administration with 9vHPV and Tdap-IPV vaccines. Serum bactericidal assays with human complement (hSBA) were used to measure anti-meningococcal antibodies against all 4 serogroups at baseline and 30 days post-vaccination. Antibodies against antigens contained in Tdap-IPV and 9vHPV were also measured. Safety data were collected within 30 days post-vaccination.

**Results:**

A total of 463 subjects were enrolled in this study. Non-inferiority between MenACYW-TT and MCV4-TT, based on seroprotection rate (percentage of subjects achieving hSBA titers ≥ 1:8), was demonstrated for all 4 serogroups one month following meningococcal vaccination. Thirty days post vaccination, hSBA geometric mean titers (GMTs) were higher for MenACYW-TT vs MCV4-TT for serogroups C, Y and W, and comparable for serogroup A. The percentages of subjects with an hSBA vaccine seroresponse were higher for MenACYW-TT vs MCV4-TT for all 4 serogroups **(Table)**. When MenACYW-TT was administered alone or concomitantly with 9vHPV and Tdap-IPV vaccines, the immunogenicity results were comparable or higher in subjects receiving MenACYW-TT alone than in those receiving all vaccines concomitantly. The safety profiles were comparable between MenACYW-TT and MCV4-TT, administered alone or concomitantly with 9vHPV and Tdap-IPV vaccines, with no safety concerns observed during this study.

**Figure:**
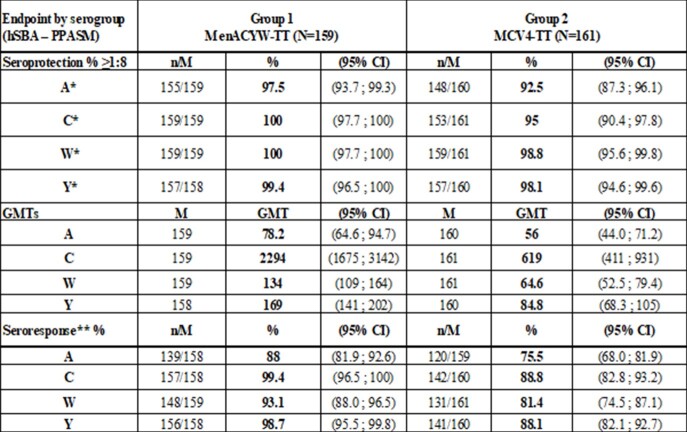

**Conclusion:**

The trial met the primary objective. MenACYW-TT induced non-inferior immune responses to all serogroups based on seroprotection rates, and higher or comparable GMTs and seroresponse rates compared to MCV4-TT.

**Disclosures:**

**Céline Zocchetti, MSc**, Sanofi: Stocks/Bonds **Olga Syrkina, MD**, Sanofi: Stocks/Bonds **Siham Bchir, MSc**, Sanofi: Sanofi's Employee|Sanofi: Stocks/Bonds **Isabelle Bertrand-Gerentes, MD**, Sanofi: Stocks/Bonds

